# A Plea for More Pelvimetry

**Published:** 1914-03

**Authors:** Frank Giuffrida

**Affiliations:** Atlanta, Ga.; Out-Patient Department, Atlanta Medical College


### A PLEA FOR MORE PELVIMETRY.

Frank Giuffrida, M. D., Atlanta, Ga.

OUT-PATIENT DEPARTMENT, ATLANTA MEDICAL COLLEGE.

It has not been so long that pelvimetry was first begun by some of the leading obstetricians over the country, and became universal among the teachers of obstetrics. I would hate to think that there is any school of medicine, be it ever so small, that does not require its students to make or become familiar with pelvimetry. When we look back and see what could have been done had we measured the pelvis of some poor woman who is dead and almost forgotten, it makes a creepy feeling go through us, if we have an obstetrical conscience. Even the carpenters who do good work will not trust to luck. They use calipers and measurements on lumber before cutting it, while we guess the pelvic measurements of a woman who is about to enter a hard ordeal. She could expect such treatment from a granny-woman, but we must not allow ourselves to come to this.

The greatest obstetricians on earth do pelvimetry on their patients, and do it thoroughly. Without it we are running too much risk. How do we know what will happen where so many possibilities exist, especially in the primapara? How many times have you heard of a man pulling with forceps, with another man, perhaps two, pulling on him from behind, and even then having to accomplish delivery by accidental or some other operation which is destructive to both mother and child? It does not take very much time away from the busy practitioner to do pelvimetry, and then you have the measurements of the patient for any subsequent labors.

If you follow obstetrics regularly you will some day bump up against a badly contracted pelvis which will make you wish you could do, or had done pelvimetry. You may not have this experience soon, but you are bound to have it some day. Pelvic deformities are by no means rare. I make it a practice to measure every primapara that I deliver, regardless of whether it is a charity or wealthy patient. Every woman has a right to expect this much precaution on our part, none of us have become so expert that we can guess her measurements. The pelvimeter is not an expensive instrument and takes up but little room in the obstetrical kit. We find it important to make anti-partum examinations. Why do we make them if we are not trying to find out the condition of the pelvis? How will you know, guess at it? Guessing is not practiced by Edgar, Williams, DeLee, Hirst, or any other competent man. If they find it necessary to do pelvimetry, I think we little fellows should as much as try. It only takes a few times to get the habit. Abnormal pelves take on so many different shapes that they are hard to classify. Don't try any more guessing on the pelvis, get a pelvimeter, it will pay for itself hundreds of times in your practice. The importance of pelvimetry should be instilled into every general practitioner's mind, as he is liable to trust to his forceps just a little more than the man who is doing obstetrics almost exclusively.

I believe the profession will agree with me when I say that we see many women delivered by forceps who should have been delivered by celio-hysterotomy. Pelvimetry has a great field of usefulness and paves the way to better obstetrics. The pelvimeter has both scales for measurement and is easy to manipulate. You can use it for estimating the size of the fœtal head in utero (Cephalometry), and, after awhile, you may become expert in making measurements. Should you find contractions or anomalies of any kind, remember the least possible diameter of the pelvis through which a living child can be delivered with or without forceps.

